# Validation of a Questionnaire Assessing Pregnant Women’s Perspectives on Addressing the Psychological Challenges of Childbirth

**DOI:** 10.3390/nursrep15010008

**Published:** 2024-12-31

**Authors:** Mihaela Corina Radu, Mihai Sebastian Armean, Razvan Daniel Chivu, Justin Aurelian, Melania Elena Pop-Tudose, Loredana Sabina Cornelia Manolescu

**Affiliations:** 1 Department of Microbiology, Parasitology and Virology, Faculty of Midwives and Nursing, “Carol Davila” University of Medicine and Pharmacy, 020021 Bucharest, Romania; loredana.manolescu@umfcd.ro; 2 Emergency Hospital County, Str. Mihai Bravu no 106, 100409 Ploieşti, Romania; 3 Department of Nursing, Faculty of Midwifery and Nursing, “Carol Davila” University of Medicine and Pharmacy, 020021 Bucharest, Romania; 4 Department of Pharmacology, Toxicology and Clinical Pharmacology, Faculty of Medicine, “Iuliu Hateganu” University of Medicine and Pharmacy, Str. Victor Babes, no. 8, 400347 Cluj Napoca, Romania; sebastian.armean@umfcluj.ro; 5 Department of Public Health and Health Management, Faculty of Midwifery and Nursing, “Carol Davila” University of Medicine and Pharmacy, 050474 Bucharest, Romania; razvan.chivu@umfcd.ro; 6 “Prof Dr. Th. Burghele” Clinical Hospital Bucharest, 061344 București, Romania; 7 Department of Obstetrics and Gynecology, Faculty of Midwives and Nursing, “Carol Davila” University of Medicine and Pharmacy, 020021 Bucharest, Romania; melania.tudose@umfcd.ro

**Keywords:** questionnaire, pregnant women, midwives, childbirth education, nursing

## Abstract

Introduction: Pregnant women’s experiences and concerns regarding childbirth are complex, necessitating a multidimensional and personalized approach in maternal care. This study explores the psychological and emotional factors influencing pregnant women’s decisions regarding their mode of delivery. The results will provide valuable insights for the development of educational and counseling strategies designed to support pregnant women in making informed and conscious decisions about their childbirth. Material and method: This cross-sectional study aimed to develop and validate a questionnaire exploring the psychological dimensions of childbirth. Factor analysis was employed to assess emotional perceptions, perceived medical risks, and the impact of cesarean section on pregnant women. The questionnaire was distributed online via Google Forms, using social networks like Facebook and Instagram to ensure rapid and broad accessibility. The questionnaire was available for seven months, from January to July 2023. Results: McDonald’s ω, Cronbach’s α, average inter-item correlation, and total item correlations were calculated to assess the consistency of the questionnaire items in measuring the same construct. The three-factor model emerged as the primary structure based on exploratory and confirmatory factor analyses (EFA and CFA). The first profile, centered on the psychological and emotional benefits of vaginal birth, highlights the importance of the natural birth experience for the mother’s psychological well-being. The second profile addresses concerns about medical risks and the need for interventions. The third profile focuses on perceptions and concerns related to the intelligence and adaptability of children born by cesarean section and the effects of anesthesia. Conclusions: Each profile reflects different strategies for seeking control and security amid childbirth uncertainties. These include emphasizing the psychological benefits of vaginal birth, addressing medical risks, and focusing on the impact of interventions on child development. Understanding these variables is essential for providing appropriate counseling and psychosocial support, thereby optimizing the birth experience and promoting the health of both mother and child. The integration of multi-factor and single-factor models in the questionnaire analysis serves complementary purposes, providing distinct yet interrelated insights into the instrument’s structure and validity.

## 1. Introduction

Pregnant women’s concerns and experiences regarding childbirth are a central aspect of maternal care, demanding a nuanced and personalized approach. While beneficial in certain cases, medical interventions during childbirth carry risks for both mother and child. Understanding these risks is essential, as they shape medical decisions and perceptions of childbirth [1,2,3,4].

Negative childbirth experiences significantly impact maternal psychology. Fear of childbirth can stem from the mother’s perception of the event itself. The feeling of losing control, intense pain, or perceived inadequate care can contribute to this fear, which may persist and influence decisions about future pregnancies [2]. It is estimated that 6–10% of all pregnant women experience a severe fear of childbirth [5,6,7]. This fear can overshadow the entire pregnancy, complicate labor, and lead to an increased number of cesarean sections [8,9,10,11].

Childbirth is a profound and unique experience, shaped by each woman’s social, cultural, and familial context [12,13]. Personal beliefs and expectations about childbirth are influenced by education, traditions, past experiences, and shared stories. Experiences and expectations related to pregnancy and childbirth are varied and ambivalent, often combining feelings of joy and confidence with anxiety, fears, and uncertainties. Even in the context of safe and well-developed maternal care systems, as found in high-income countries, fear of childbirth remains a common issue, affecting women’s mental and physical health before, during, and after childbirth [14,15].

This fear has repercussions on multiple levels, including on the woman’s relationships with her newborn, partner, and family [16,17,18]. Studies suggest that fear of childbirth can lead to an increase in requests for cesarean sections, as women seek to maintain control in a situation perceived as vulnerable and unsafe. Additionally, fear of childbirth can affect the quality of the emotional bond between mother and child and may cause difficulties in physical and emotional recovery postpartum [19,20,21].

However, there is a lack of consensus on the definitions related to “fear of childbirth”. The concept seems to be used as a broad label for a range of anxieties and fears that women experience regarding pregnancy and childbirth. In the specialized literature, the term has been approached in various ways, from moderate anxiety to severe anxiety disorders or even phobias. For example, “tokophobia” is defined as a pathological fear of childbirth, which may lead to complete avoidance of pregnancy or requests for surgical procedures to avoid natural labor [22,23,24].

Research on fear of childbirth identifies several key directions for understanding and addressing this issue. Although fear of childbirth is a common and recognized problem, addressing it requires a comprehensive strategy that targets both prenatal education and psychological and social support for pregnant women. Such an approach should include the development of customized prenatal education curricula focused on the specific fears women have.

Personalized prenatal education fosters security and fulfillment, supporting women in adapting to their maternal role. It helps develop essential resources for a smoother transition to motherhood. Utilizing the potential of prenatal education to address and reduce fear of childbirth requires an integrative and individualized approach. By identifying and exploring fears, personalizing educational curricula, and supporting emotional well-being, women can benefit from a less stressful and more empowering childbirth experience. This approach can improve both women’s mental health and the overall quality of maternal care.

This study takes an in-depth, multidimensional approach to the fear of childbirth, focusing on the psychological and emotional factors shaping pregnant women’s perceptions. While previous studies have generally addressed the fear of childbirth, our study places particular emphasis on the validation of a personalized prenatal education tool aimed at reducing this fear. This study is novel in integrating educational and counseling approaches tailored to pregnant women’s fears, aiming to enhance their childbirth experience and postnatal mental health. By focusing on personalized interventions, this study contributes to the development of targeted educational strategies that address the individual needs of pregnant women.

The aim of this research is to develop and validate a questionnaire to identify and understand the psychological and emotional factors that influence pregnant women’s decisions regarding their mode of delivery, in order to develop educational and counseling strategies that support informed and conscious decision-making in this complex process.

## 2. Materials and Methods

### 2.1. Study Design

A cross-sectional validation study was conducted on a sample of 1301 pregnant women who registered on digital platforms, such as forums, discussion groups, or social networks, in Romania. The study developed and validated a personalized questionnaire to explore the psychological and emotional factors influencing pregnant women’s perceptions of childbirth. This multidimensional study integrates educational and counseling approaches to better understand and address pregnant women’s fears and expectations, enhancing their childbirth experience and postnatal mental health. The study relies on factorial analysis to identify the main dimensions of pregnant women’s perceptions, such as emotional perceptions, perceived medical risks, and the impact of cesarean sections on them. This descriptive and exploratory study aims to identify psychological and emotional factors, while evaluating the effectiveness of a personalized prenatal education tool to reduce fear of childbirth and improve the mental health of pregnant women.

### 2.2. Participants

Participants were recruited by sharing an online questionnaire link on popular platforms such as Facebook, Instagram, and forums for pregnant women in Romania. The participants were invited to voluntarily complete the questionnaire, with assurance of anonymity and confidentiality of their responses.

Data collection was conducted exclusively online, ensuring accessibility and eliminating geographical barriers. The participants completed the questionnaire autonomously, without the researchers’ intervention, thus ensuring an objective and standardized data collection process.

The selection criteria for participants included a minimum age of 18 years, Romanian citizenship, the absence of serious chronic illnesses, and no history of infertility. Exclusion criteria included being under 18, the presence of any risk factor necessitating medically indicated cesarean risks (e.g., placenta previa, preeclampsia, malpresentation of the fetus), absence of pregnancy, or incomplete/contradictory data. This methodology aimed to capture diverse psychological experiences and challenges, reflecting the population’s variability.

Quality control involved verifying the collected data to ensure completeness and consistency. Incomplete or contradictory responses were excluded. To minimize errors and response biases, the questionnaire was pre-tested on a small sample of participants to validate the questions and ensure their clarity and coherence. Statistical analyses, including factorial analysis, were conducted to evaluate the instrument’s validity, reliability, and internal consistency.

Sample size calculation followed the specialized literature recommendations, requiring a minimum of five respondents per questionnaire item for valid results [25,26].This approach is commonly used in psychometric studies to ensure that the statistical analysis has sufficient power to detect significant relationships between variables, allowing researchers to evaluate the internal consistency of the questionnaire and to conduct validity tests, such as construct and content validity, with a reduced margin of error.

### 2.3. Questionnaire Administration

This study targeted pregnant women across Romania, including various counties and the capital city. The participants received an electronic announcement with an online link, distributed on digital platforms such as forums, discussion groups, or social networks such as Facebook and Instagram, inviting them to participate in a study dedicated to researching aspects of childbirth management and reproductive health in Romania. Participants accessed the link provided and decided to proceed after reading a brief description of the study, which included safety and anonymity guaranteed. Informed consent for data collection was expressed through the participants’ decision to proceed after reviewing this information. Additionally, a dedicated email address was provided for questions or clarifications.

Administered via Google Forms, the questionnaire was available online for seven months, from January to July 2023, ensuring rapid distribution and broad accessibility.

The questionnaire was structured into four distinct sections:

General Data Section: This section includes five single-response questions to collect demographic information (age, education, marital status, place of residence, occupational level).

Obstetrical Data Section: Comprising five single-response questions, this section collects information on obstetrical history, including pregnancy trimester, prior miscarriages, previous births, prenatal consultations, and preferred place of delivery.

Knowledge and Information Section: This section consists of eight questions to assess the extent to which pregnant women have been informed about pregnancy, childbirth, and postnatal care, as well as three questions concerning the importance of midwifery in antenatal and postnatal care. Specific questions included: 1. Has the doctor monitoring your pregnancy informed you about the childbirth process? 2. Has the doctor monitoring your pregnancy informed you about the importance of early contact with the baby? 3. Has the doctor monitoring your pregnancy informed you about the importance of the “golden hour”/the mother–child bonding in the first hours after birth? 4. Has the doctor monitoring your pregnancy informed you about breastfeeding and its benefits? 5. Have you participated in childbirth education courses or programs? 6. Did you find the things you learned useful (for pregnancy/labor/childbirth/postpartum)? 7. From what sources do you obtain information regarding pregnancy, childbirth, and breastfeeding? 8. Are you aware of the services that midwives can offer during pregnancy, childbirth, and the postpartum period? 9. In your opinion, would access to a midwife during pregnancy, childbirth, and the postpartum period be helpful? 10. On a scale from 1 to 10 (where 1 is the least important and 10 is very important), how important do you consider the role of the midwife in the stages of pregnancy, childbirth, and postpartum? 11. What expectations do you have from the medical staff during childbirth?

Attitudinal Statements Section: Pregnant women were asked to express their agreement, disagreement, or neutrality on the following 22 statements regarding vaginal birth and cesarean section. For scoring, a response of “agreement” was given a score of 3 points, “disagreement” 1 point, and “neutral” 2 points. Higher scores, calculated by summing the points for each response, indicate a better understanding of childbirth-related aspects, including the benefits and risks associated with vaginal birth and cesarean section. The statements are as follows:

A1—Childbirth is a physiological process that should only be intervened in when medically necessary.
A2—Skin-to-skin contact, mother and child, is easier to perform with vaginal birth.
A3—After a vaginal birth, the mother’s recovery is easier and faster.
A4—Vaginal birth increases a woman’s self-confidence.
A5—The emotional bond between mother and child is stronger after a vaginal birth.
A6—Scheduled cesarean sections are associated with the risk of prematurity for the newborn.
A7—Children born through vaginal delivery adapt better to life outside the womb.
A8—Fear of birth pain influences the choice of delivery method.
A9—Cesarean section on request is more accessible in private healthcare.
A10—It is the pregnant woman’s right to choose the delivery method, but her choice should be informed and medically advised.
A11—Cesarean section is a lifesaving intervention for mother and child when there are complications during pregnancy and childbirth.
A12—Pain during childbirth is one of the indications for a cesarean section.
A13—The medications used for anesthesia in a cesarean section are harmful to the fetus.
A14—Vaginal birth decreases sexual satisfaction.
A15—Children born by cesarean section are healthier than those born vaginally.
A16—The baby’s lack of adaptation to life outside the womb is more frequent with cesarean sections.
A17—Children born by cesarean section are more intelligent.
A18—Vaginal birth is preferable because the scars left after a cesarean section are unsightly.
A19—Proper information and education for pregnant women regarding cesarean sections could lead to more conscious and informed choices.
A20—Cesarean section should be an available option for all pregnant women.
A21—Water birth can help reduce the need for pain medication and medical interventions.
A22—Medical interventions (such as induced labor, episiotomy, and cesarean section) during childbirth are overused and should be avoided when possible.

The development and validation of the questionnaire were carried out in four distinct stages, each playing an essential role in ensuring the instrument’s relevance and reliability.

The first stage involved an extensive review of the specialized literature to identify common psychological challenges faced by pregnant women. A variety of studies and publications were analyzed, focusing on the impact of pregnancy on women’s mental and emotional health, as well as on the psychological factors that can influence this period.

Databases such as PubMed, Scopus, and Google Scholar were utilized to identify relevant articles. The search employed keywords such as “fear of childbirth”, “tokophobia”, “perceptions of vaginal birth”, “benefits of cesarean section”, and “psychological factors in pregnancy”. Methodologically relevant studies were selected. Based on the analyzed literature, the items for the questionnaire were developed [27,28,29,30,31,32,33,34,35,36,37,38,39,40,41,42,43,44,45,46,47,48,49,50,51,52,53,54,55].

Based on the information gathered from the literature, the questionnaire items were developed with a focus on relevant psychological aspects. The items were created to cover a wide range of challenges.

A preliminary version of the questionnaire was tested on a small group of pregnant women in a pilot test. This test allowed for the assessment of item clarity and the identification of any potential issues related to understanding or interpreting the questions. Participant feedback during the pilot test was instrumental in refining and improving the questionnaire.

During the final stage, the refined questionnaire was distributed to a larger sample of pregnant women for validation. This stage included statistical analysis of the data to verify the internal consistency, reliability, and validity of the questionnaire.

### 2.4. Statistical Analysis

The questionnaire data were analyzed using Microsoft Office Excel and IBM^®^ SPSS^®^ Statistics Version 23.0. Data processing utilized the COUNTIFS function in Excel to filter and sort the initial database. Statistical analysis was performed using JASP 0.18.3 R © JASP Team (2024), JASP (Version 0.18.3) [Computer software], and R version 4.3.3 (Copyright © 2024 The R Foundation for Statistical Computing, R Core Team (2024). R: A language and environment for statistical computing. R Foundation for Statistical Computing, Vienna, Austria), using the following packages: EFA.dimensions [56], EFAtools [57], lavaan [58], semPlots [59], and gtsummary [60]. The study’s significance level, α, was set at 0.05, meaning that *p*-values below 0.05 were considered statistically significant.

Descriptive statistics detailed the socio-demographic characteristics of the sample offering a comprehensive overview of the participant group’s structure. Quantitative variables, such as age or hospital stay duration, were summarized using the mean, and standard deviations were used to highlight central tendency and variability. Qualitative variables, including gender, education level, and marital status, were reported as frequencies and percentages to illustrate their distribution within the sample. This type of descriptive analysis is essential for understanding the social and demographic context in which the questionnaire was applied, allowing researchers to evaluate the sample’s diversity and representativeness.

To evaluate the questionnaire’s structure and identify relevant psychological constructs measured, an exploratory factor analysis (EFA) was conducted. This method identified latent factors influencing responses and organized items into coherent groups, each representing a distinct dimension of the studied construct. Thus, the EFA enabled the identification of hidden structures within the data and the theoretical validation of the questionnaire.

Using the five-point Likert scale responses, a correlation matrix was calculated to assess inter-item relationships. Eigenvalues were then established to determine the optimal number of factors to extract. Eigenvalues quantified the variance explained by each factor, aiding in the identification of those that significantly contributed to explaining response variation.

A scree plot and parallel analysis visualized the optimal number of factors, identifying the ‘elbow’ point where additional factors contributed minimally to variance. This technique aids researchers in avoiding the extraction of an excessive number of factors and selecting only those with clear theoretical and statistical significance.

EFA utilized the maximum likelihood extraction method combined with Varimax rotation and Kaiser normalization to enhance clarity and interpretability of the factors. Varimax rotation maximizes variability between the obtained factors, making each factor as distinct as possible, which facilitates interpretation.

Factors were selected based on factor loadings and interpretability, ensuring both statistical significance and theoretical relevance. This ensured that the extracted factors were not only mathematically significant but also theoretically relevant, representing coherent and applicable psychological constructs. This process allowed for the refinement and validation of the questionnaire as an effective tool for measuring the targeted constructs.

### 2.5. Ethical Approval

The Ethics Committee of Dr. Constantin Andreoiu Emergency Hospital, Ploiești, Romania (41482, 9 August 2022) approved the study, which complied with the ethical standards of the Declaration of Helsinki. Participation was contingent on obtaining informed consent.

## 3. Results

### 3.1. Consistency Analysis of the Questionnaire

The socio-demographic and obstetric analysis shows that half of the participants, 689 (52.95%), were aged 18–29 years, and most, 1060 (81.47%), were married. Of our group of 1301 pregnant women, three-quarters (74.78%) had higher education and a regular job (75.86%). The majority of survey participants, 936 (71.94%), lived in an urban area, one-third (36.58%) had attended childbirth education classes, and more than half (60.79%) were in their third trimester of pregnancy. A total of 298 (22.9%) respondents expressed a preference to avoid hospital births, while 347 (26.67%) did not emphasize the importance of control during the birth process.

To evaluate the extent to which the items (questions) in the questionnaire consistently measure the same construct or concept, McDonald’s ω, Cronbach’s α, the average inter-item correlation, and each item’s correlation with the total questionnaire score were calculated. The results for the initial 22-item questionnaire are presented in Table 1 and Table 2 (scores for A22, A21, A19, and A18 were reversed for consistency: 1 -> 3, 2 -> 2, 3 -> 1).

McDonald’s ω (0.802) and Cronbach’s α (0.787) indicate good internal consistency, with narrow confidence intervals reflecting stability and precision.

It was observed that not all items had the optimal correlation (e.g., >0.20) with the total score; therefore, the following items were removed: A10, A11, A12, A15, A18, A19, A20, A21. The new metrics are presented in the following tables:

Both McDonald’s ω and Cronbach’s α demonstrate high internal consistency reliability, indicating that the measurement instrument’s items are homogeneous and effectively measure the same underlying construct (Table 3).

Most of the items contribute positively to the internal consistency of the scale (Table 4).

### 3.2. Exploratory Factor Analysis

Next, a random subsample of 399 observations was drawn from the initial sample of 1301 observations for an exploratory factor analysis (EFA). The entire sample was then used for a confirmatory factor analysis (CFA), utilizing the results obtained from the EFA.

The primary goal of the exploratory factor analysis (EFA) was to simplify the dataset by identifying latent factors that explain the correlations among observed variables. In the first stage, the optimal number of factors was determined using a polychoric item correlation matrix and the following tests: the Kaiser empirical criterion (EMPKC), the HULL method, and parallel analysis, which compares the eigenvalues of the item correlation matrix with the eigenvalues of simulated matrices (100 polychoric correlation matrices were simulated).

The optimal number of factors was found to be three for EMPKC, one for the HULL method, and three for parallel analysis. Given that the number of factors did not coincide, it was decided to proceed with an analysis determining item loadings on three theoretical factors, while the confirmatory factor analysis would compare the two models and decide which is more appropriate for our study data.

The factor extraction method used was PAF (principal axis factoring—common factor analysis), and the rotations used for optimal loading extraction were oblique (promax, oblimin, quartimin, simplimax, bentlerQ, geominQ). Items 7 and 14 were excluded from the questionnaire as their factor loadings were below the 0.40 threshold on all three factors (Table 5 with promax rotation).

Factor loadings and uniqueness analyses revealed that Factor 1 represents positive experiences with vaginal birth. Factor 2 encompasses medical and decision-making aspects, while Factor 3 lacks clear definition in this dataset. The item uniqueness suggests that certain items have significant unexplained variability, indicating the need for further analysis to clarify the measurements. The consistency analysis of the questionnaire is resumed with items 7 and 14 removed (Table 6).

Both McDonald’s ω and Cronbach’s α show acceptable internal consistency values for the scale used. These values indicate that the scale items are well-correlated with each other and that the scale is reliable for measuring the targeted construct. The narrow confidence intervals suggest that the estimates are precise and stable.

It can be observed that items A4 (0.535), A8 (0.533), and A9 (0.517) have high correlations (above 0.5). These items are strongly correlated with the total scale score, indicating a significant contribution to the internal consistency of the scale and are essential for measuring the targeted construct. Items A1 (0.487), A3 (0.405), A5 (0.457), A16 (0.396), A2 (0.305), and A13 (0.317) have moderate correlations (between 0.3 and 0.5). These items are well-aligned with the rest of the scale and contribute to internal consistency, though not as strongly as those with higher correlations. They are adequate, but there is potential for improvement to increase internal consistency. Items A6 (0.241), A17 (0.249), and A22 (0.230) have low correlations (below 0.3), suggesting that they do not correlate well with the rest of the scale. These items may be measuring different aspects of the construct or may be worded in a way that is not well understood by respondents (Table 7).

Most items have moderate or high correlations with the total scale score, indicating an acceptable level of internal consistency. Items with high correlations are particularly useful for measuring the construct.

In the exploratory factor analysis to determine the number of factors using the previous procedure, the results were identical to those obtained with the questionnaire including A7 and A14 (Table 8).

**Figure 1:**
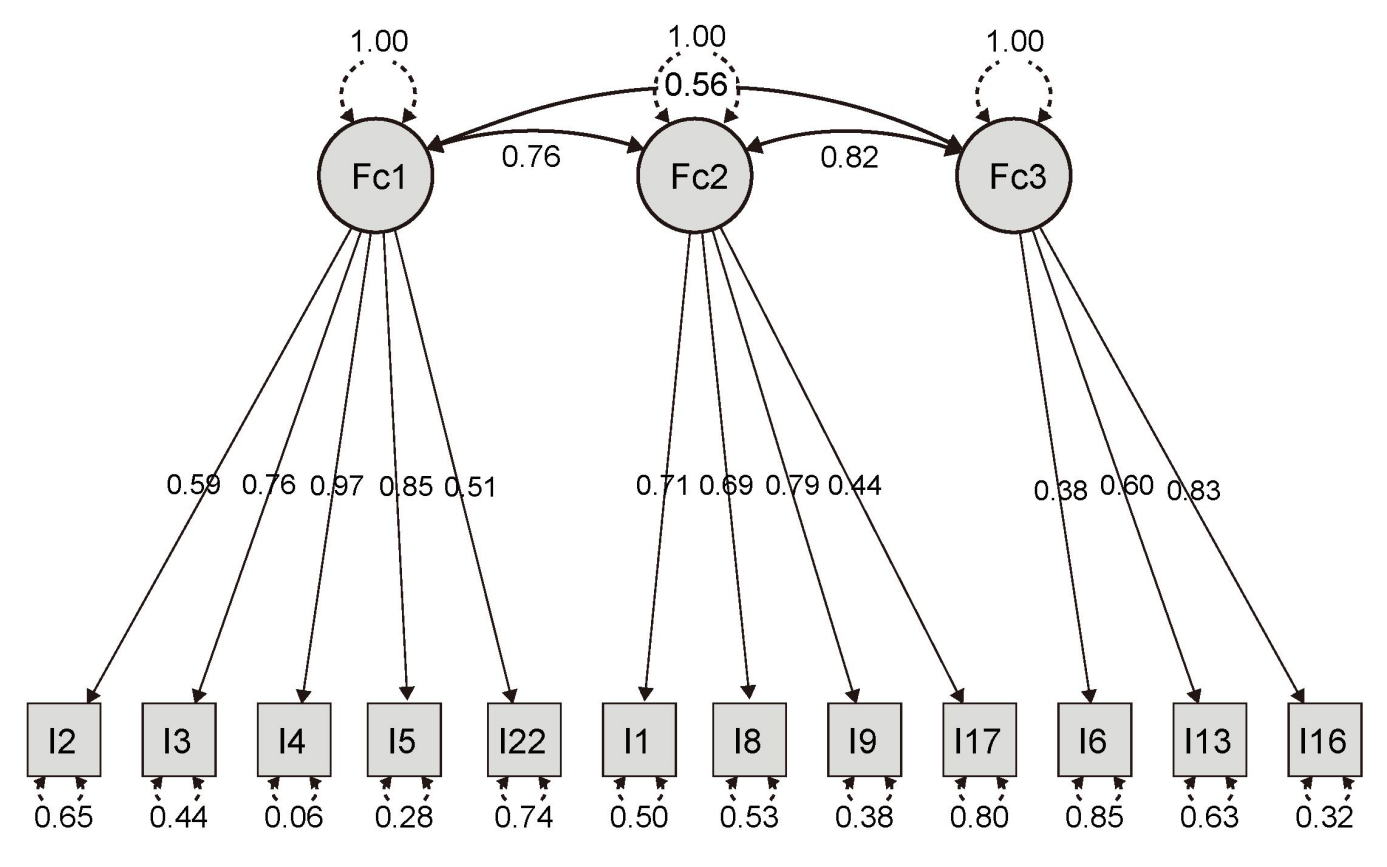
Scree plot model.

The model is optimal, with all loadings above 0.40 and uniqueness over 0.40 for each item (Figure 1).

The exploratory factor analysis reveals three latent variables: F1 with items 2, 3, 4, 5, and 22; F2 with items 1, 8, 9, and 17; and F3 with items 6, 13, and 16. The proportion of variance explained by these three factors is presented in the following table:

In the un-rotated solution, Factor 1 accounted for the largest proportion of the total variance, with the remaining factors contributing much less. This dominance of Factor 1 highlights the need for rotation to achieve a more balanced redistribution of variance, which enhances interpretability. Post-rotation, the total variance explained remains unchanged, but it is more evenly distributed across factors, facilitating clearer interpretation. This approach makes the interpretation of factors clearer and more useful, as each factor now explains a significant and distinct portion of the total variance (Table 9).

### 3.3. Confirmatory Factor Analysis

Confirmatory factor analysis (CFA) validates predefined theoretical models by testing relationships between observable variables and latent factors. Unlike exploratory factor analysis (EFA), which explores the data structure without prior assumptions, CFA is used to confirm a specific factor structure based on theory or previous results.

The model used was the one determined in the exploratory factor analysis, with the estimator being DWSL (diagonalized weighted least square). The results are presented in Table 10.

Factor 1 is defined primarily by items A4 and A5, which show strong loadings, emphasizing their critical role in representing the construct associated with this factor.

Factor 2 is characterized by a very strong loading from item A9 and strong contributions from items A1 and A8, highlighting their significance for this factor.

Factor 3 is primarily represented by item A16, underscoring its centrality to this factor.

All factor loadings are highly significant (*p* < 0.001), affirming that each item meaningfully contributes to its designated factor. The minimal standard errors and tight confidence intervals reflect high precision in the estimation of factor loadings.

This analysis shows that the items are well-correlated with their corresponding factors, indicating a solid and reliable factor structure.

The factor variances were standardized to 1.000 for all three factors, a common practice in factor analysis to simplify the interpretation and comparison of loadings. The zero standard errors and fixed confidence intervals indicate that these variances were predefined rather than estimated from the data. Fixing the variances at 1.000 allows the factor loadings to be interpreted in standardized terms, which simplifies comparisons between factors and across different analyses (Table 11).

The covariances between Factor 1 and Factor 2 (0.763) and between Factor 2 and Factor 3 (0.616) indicate strong and moderately strong correlations between these factors. This suggests that these factors share a significant portion of their variance and may measure related aspects of the studied construct (Table 12).

The moderate covariance between Factor 1 and Factor 3 (0.562) signifies a meaningful but comparatively weaker relationship than the other factor pairs.

The statistically significant covariances (*p* < 0.001) confirm that the relationships between factors are robust and unlikely to result from chance. The narrow confidence intervals and small standard errors indicate that the covariance estimates are precise and reliable.

We observe that only one loading was below 0.40, which was for item A6 on factor 3, with all coefficients having statistically significant *p*-values. Additionally, there were significant correlations between the three factors (Pearson’s r between 0.56 and 0.76) (Table 13).

### 3.4. Goodness of Fit Diagnostic Tests

Goodness of fit diagnostic tests are statistical methods used to assess how well a theoretical model fits the observed data. These tests are essential in CFA, structural modeling, and other statistical methods to determine whether the specified model adequately describes the structure of the collected data.

When building a theoretical model, such as a factor model, it is assumed that the observed variables behave in a certain way in relation to the latent factors. Goodness of fit tests help evaluate how well these theoretical assumptions match reality, that is, the empirical data (Table 14).

The factor model achieved a considerably lower chi-square value (230.578) than the baseline model, highlighting a significant improvement in fit relative to the baseline. The *p*-value for the factor model is very low (<0.001), suggesting that there is a statistically significant difference between the proposed model and a perfect fit model. However, in practical contexts, complex models rarely achieve perfect fit, and this low *p*-value does not negate the usefulness of the factor model.

While the chi-square value and *p*-value indicate an imperfect fit, the significant reduction in the chi-square value from the baseline model to the factor model suggests that the factor model is significantly more suitable for the analyzed data.

The significant *p*-value (<0.001) suggests an imperfect fit; however, the large sample size inflates the chi-square statistic, reducing its reliability as a standalone measure of fit. The chi-square (χ^2^)/degrees of freedom (df) ratio of 4.52 is within the acceptable threshold of <5.00 suggested by some researchers [61], though it exceeds the more stringent cut-off of <2.00 proposed by others [62].

Key fit indices, including CFI, TLI, NNFI, NFI, IFI, and RNI, all exceed the threshold of 0.95, reflecting an excellent fit of the model to the data. The Parsimony Normed Fit Index (PNFI) of 0.761 indicates a satisfactory balance between model complexity and goodness of fit, supporting the model’s adequacy. The consistently high values of the fit indices demonstrate that the proposed model is well-structured and captures the underlying data patterns effectively (Table 15).

These results suggest that the analyzed factor model fits the data very well and is both parsimonious and representative of the latent structure of the studied variables.

An RMSEA value below 0.06 is considered an indicator of good fit [63], and a non-significant *p*-value for the RMSEA test > 0.05 is considered to denote optimal fit.

Hoelter’s critical N at α = 0.01 was 437.302, which is lower than the sample size on which the CFA was conducted (1301 observations), also indicating a good fit by this criterion.

The SRMR was below 0.08, also indicating optimal fit; the MFI and GFI additionally indicated a good fit (Table 16).

### 3.5. The Single-Factor Model (Using the Same Estimator as the Previous Model)

Items A4 (0.943), A5 (0.833), A3 (0.727), A9 (0.709), and A8 (0.619) exhibit strong or very strong correlations with Factor 1, indicating that they robustly represent the latent construct under investigation (Table 17).

Items A1 (0.624), A2 (0.566), A16 (0.573), A13 (0.441), A17 (0.396), and A22 (0.475) display moderate loadings, contributing to Factor 1 while exhibiting weaker associations compared to the strongest indicators.

Item A6 (0.290) has a weak correlation with Factor 1, indicating limited relevance to the latent construct and potential for exclusion or re-evaluation.

All *p*-values are below 0.001, indicating that all loadings are statistically significant.

Items A6 (0.916), A17 (0.843), A13 (0.805), and A22 (0.775) indicate very strong correlations with their corresponding factors, suggesting that these items are excellent indicators of the measured constructs.

Items A2 (0.679), A8 (0.617), and A1 (0.611) show strong correlations with their factors, indicating solid measurement of the respective constructs.

Items A3 (0.472), A9 (0.497), and A16 (0.672) have moderate correlations, suggesting a reasonable contribution to their factors.

Item A4 (0.111) has a low correlation (Table 18).

### 3.6. Other Diagnostic Tests

The factor model has a much lower chi-square value compared to the baseline model, indicating a significantly better fit of the factor model to the observed data.

The very low *p*-value (<0.001) for the factor model indicates a statistically significant deviation from a perfect fit. However, in practical contexts, complex models rarely achieve perfect fit, and this low *p*-value does not negate the usefulness of the factor model.

While the chi-square value and *p*-value indicate an imperfect fit, the significant reduction in the chi-square value from the baseline model to the factor model suggests that the factor model is much more suitable for the analyzed data (Table 19)

Most of the indices (CFI, TLI, NNFI, NFI, IFI, RNI) have very high values, above 0.95, indicating an excellent fit of the model to the observed data. The PNFI has a value of 0.786, indicating a reasonable parsimony of the model, which balances well between fit and complexity. The high values of these indices suggest that the proposed model is well-specified and adequately represents the data. These results suggest that the analyzed factor model fits the data very well and is both parsimonious and representative of the latent structure of the studied variables, though slightly weaker than the fit of the three-factor model (Table 20).

The RMSEA is greater than 0.06, and the *p*-value for the RMSEA test > 0.05 is statistically significant, indicating that the fit of the single-factor model is worse than that of the three-factor model. Factor analysis identified three main factors reflecting distinct profiles of pregnant women in the context of childbirth (Table 21).

## 4. Discussion

Assessing pregnant women’s perceptions of childbirth is crucial for understanding the multifaceted nature of their experiences and for developing tailored education and support strategies. Such questionnaires necessitate a robust theoretical framework and rigorous empirical validation to comprehensively capture the psychological, medical, and social dimensions of childbirth perceptions. In analyzing this questionnaire, both multi-factor and single-factor models were applied to examine its structure and validate its suitability to the studied reality.

The three-factor model was identified as optimal for this instrument, providing a detailed understanding of various aspects of the childbirth experience. However, the single-factor model was tested as part of a comparative approach to confirm the necessity and superiority of the multi-factor model. The concurrent application of multi-factor and single-factor approaches not only validates the robustness of the instrument but also ensures its practical utility in addressing the varied psychological, medical, and social concerns of pregnant women.

Factorial analysis revealed three distinct factors representing unique profiles of pregnant women concerning their perceptions of childbirth.

The integration of multi-factor and single-factor models in the questionnaire analysis serves complementary purposes, providing distinct yet interrelated insights into the instrument’s structure and validity.

The three-factor model emerged as the primary structure based on exploratory and confirmatory factor analyses (EFA and CFA). This model demonstrated a solid theoretical and empirical foundation, with factors representing:

The first profile, centered on the psychological and emotional benefits of vaginal birth, highlights the importance of the natural birth experience for the mother’s psychological well-being. Pregnant women who value this aspect may perceive vaginal birth as a confirmation of their maternal abilities and as an experience of empathy and connection with their child. This profile underscores the importance of targeted psychological and emotional support in enhancing maternal well-being and promoting a smoother postpartum recovery [64,65,66,67].

The second profile addresses concerns about medical risks and the need for interventions. Pregnant women in this profile are likely more sensitive to potential medical complications and the need for surgical or other medical procedures. This concern is often associated with a sense of uncertainty and anxiety about the birth outcome. For these women, healthcare providers must offer clear and detailed information, promptly respond to questions, and ensure continuous monitoring to reduce anxiety and promote a sense of security.

The third profile focuses on perceptions and concerns related to the intelligence and adaptability of children born by cesarean section and the effects of anesthesia. Pregnant women in this profile may express fears about the long-term impact of surgical interventions on the child’s cognitive and behavioral development. These concerns reflect an increased awareness of the potential neuropsychological implications of birth and highlight the need for well-founded information regarding the safety and efficacy of these procedures [68,69,70,71].

The three-factor model yielded excellent fit indices (CFI = 0.988, TLI = 0.985, RMSEA = 0.052, SRMR = 0.059), confirming its strong explanatory power regarding the variance in the observed data. All items showed statistically significant loadings on their respective factors, validating the multidimensional nature of the questionnaire.

The rotated solution redistributed the variance evenly among factors, improving interpretability and showing that each factor captures a unique aspect of the childbirth experience.

While the superiority of the multi-factor structure is evident, the single-factor model was evaluated to facilitate comparative validation. This approach explored whether a simplified structure could adequately explain the data or if the complexity of the three-factor model was necessary. While the single-factor model demonstrated reasonable fit indices (CFI = 0.965, RMSEA = 0.087, SRMR = 0.081), these were considerably weaker than those of the three-factor model, indicating that the simplified model cannot fully capture the multidimensional nature of the constructs. Items related to maternal confidence and psychological bonding (e.g., “Vaginal birth increases a woman’s self-confidence”) showed strong loadings, suggesting they are central to the construct. However, the reduced explanatory power highlighted the limitations of aggregating all dimensions into a single factor. The single-factor model served as a baseline to confirm that the multi-factor model significantly improves explanatory power and fit.

The multi-factor model provides a nuanced understanding of the psychological, medical, and social dimensions of childbirth perceptions. It aligns with the need for targeted interventions and individualized maternal care.

Despite its simplicity, the single-factor model highlighted the interrelation among factors, suggesting the potential relevance of a higher-order latent construct (e.g., overall perception of childbirth). However, its reduced specificity underscores the limitations of this approach in capturing nuanced dimensions.

## 5. Conclusions

The factor analysis identified three distinct profiles that reflect essential aspects of the childbirth process, offering a deeper understanding of the variability in pregnant women’s perceptions and attitudes. These findings emphasize the importance of adopting a personalized approach in birth planning and management. Each profile highlights a unique set of needs and expectations that healthcare providers must address. Across the identified profiles, a central concern is the focus on the safety and well-being of both the mother and child, expressed through the desire for informed decision-making and appropriate support during childbirth.

Each profile distinctly represents a way of seeking control and security in response to the uncertainties and risks associated with childbirth, including the psychological benefits of vaginal birth, concerns about medical risks, or apprehensions regarding the impact of interventions on child development. Understanding these variables is essential for providing tailored counseling and psychosocial support, thereby enhancing the childbirth experience and promoting the health of both mother and child. This holistic approach, which recognizes and respects the diversity of pregnant women’s experiences, can significantly improve the quality of maternal care and foster a positive and responsible transition to motherhood.

Testing both models confirmed the robustness of the three-factor structure as the optimal framework for this questionnaire. The process underscores the importance of capturing the complexity of pregnant women’s perspectives on childbirth, ensuring that interventions and educational strategies comprehensively address diverse needs. This finding validates the instrument’s ability to measure distinct yet interconnected dimensions of the maternal experience.

The questionnaire can be integrated into clinical practice to enhance the care of pregnant women and improve their childbirth experiences. It facilitates the identification of psychological and emotional profiles of patients, such as fear of childbirth (tokophobia) or concerns about medical risks. Based on the responses, personalized care plans can be developed, including specific prenatal education, tailored psychological counseling, and appropriate medical guidance.

The questionnaire results can facilitate discussions between patients and healthcare providers regarding childbirth options, ensuring alignment between personal preferences and medical recommendations. Postpartum application of the instrument allows for the evaluation of patient satisfaction and the extent to which their needs and expectations were met, contributing to the improvement of future maternal care services.

## Figures and Tables

**Table 1:** Questionnaire consistency analysis.

Estimate	McDonald’s ω	Cronbach’s α
Point Estimate	0.802	0.787
Lower Limit of 95% Confidence Interval	0.787	0.770
Upper Limit of 95% Confidence Interval	0.818	0.802

**Table 2:** Item correlations with final score.

Item	Item-Rest Correlation
A1—Childbirth is a physiological process that should only be intervened in when medically necessary.	0.430
A2—Skin-to-skin contact, mother and child, is easier to perform with vaginal birth.	0.421
A3—After a vaginal birth, the mother’s recovery is easier and faster.	0.495
A4—Vaginal birth increases a woman’s self-confidence.	0.695
A5—The emotional bond between mother and child is stronger after a vaginal birth.	0.638
A6—Scheduled cesarean sections are associated with the risk of prematurity for the newborn.	0.242
A7—Children born through vaginal delivery adapt better to life outside the womb.	0.585
A8—Fear of birth pain influences the choice of delivery method.	0.476
A9—Cesarean section on request is more accessible in private healthcare.	0.550
A10—It is the pregnant woman’s right to choose the delivery method, but her choice should be informed and medically advised.	0.047
A11—Cesarean section is a lifesaving intervention for mother and child when there are complications during pregnancy and childbirth.	0.055
A12—Pain during childbirth is one of the indications for a cesarean section.	0.112
A13—The medications used for anesthesia in a cesarean section are harmful to the fetus.	0.340
A14—Vaginal birth decreases sexual satisfaction.	0.455
A15—Children born by cesarean section are healthier than those born vaginally.	0.180
A16—The baby’s lack of adaptation to life outside the womb is more frequent with cesarean sections.	0.427
A17—Children born by cesarean section are more intelligent.	0.196
A18—Vaginal birth is preferable because the scars left after a cesarean section are unsightly.	0.073
A19—Proper information and education for pregnant women regarding cesarean sections could lead to more conscious and informed choices.	0.178
A20—Cesarean section should be an available option for all pregnant women.	0.061
A21—Water birth can help reduce the need for pain medication and medical interventions.	0.196
A22—Medical interventions (such as induced labor, episiotomy, and cesarean section) during childbirth are overused and should be avoided when possible.	0.381

**Table 3:** Consistency analysis of the questionnaire after removing items A10, A11, A12, A15, A18, A19, A20, and A21.

Estimate	McDonald’s ω	Cronbach’s α
Point Estimate	0.836	0.829
Lower Limit of 95% Confidence Interval	0.823	0.816
Upper Limit of 95% Confidence Interval	0.849	0.842

**Table 4:** Item correlations with the final score after removing items A10, A11, A12, A15, A18, A19, A20, and A21.

Item	Item-Rest Correlation
A1—Childbirth is a physiological process that should only be intervened in when medically necessary	0.454
A2—Skin-to-skin contact, mother and child, is easier to perform during vaginal birth	0.421
A3—After a vaginal birth, the mother’s recovery is easier and faster	0.487
A4—Vaginal birth increases a woman’s self-confidence	0.717
A5—The emotional bond between mother and child is stronger after a vaginal birth	0.682
A6—Scheduled cesarean sections are associated with the risk of prematurity for the newborn	0.201
A7—Children born through vaginal delivery adapt better to life outside the womb	0.625
A8—Fear of birth pain influences the choice of delivery method	0.509
A9—Elective cesarean sections are more accessible in private healthcare	0.563
A13—The medications used for anesthesia in cesarean sections are harmful to the fetus	0.346
A14—Vaginal birth leads to a decrease in sexual satisfaction	0.437
A16—The baby’s lack of adaptation to life outside the womb is more frequent in cesarean sections	0.443
A17—Children born by cesarean section are more intelligent	0.257
A22—Medical interventions (such as induced labor, episiotomy, cesarean section) are excessively used and should be avoided if possible	0.331

**Table 5:** Factor loadings after promax rotation.

Item	Factor			
	1	2	3	Uniqueness
A1—Childbirth is a physiological process that should only be intervened in when medically necessary		0.531		0.64
A2—Skin-to-skin contact, mother and child, is easier to perform during vaginal birth	0.497			0.757
A3—After a vaginal birth, the mother’s recovery is easier and faster	0.485			0.7
A4—Vaginal birth increases a woman’s self-confidence	0.649			0.425
A5—The emotional bond between mother and child is stronger after a vaginal birth	0.732			0.433
A6—Scheduled cesarean sections are associated with the risk of prematurity for the newborn			0.512	0.746
A7—Children born through vaginal delivery adapt better to life outside the womb	0.363			0.647
A8—Fear of birth pain influences the choice of delivery method		0.575		0.559
A9—Elective cesarean sections are more accessible in private healthcare		0.488		0.549
A13—The medications used for anesthesia in cesarean sections are harmful to the fetus			0.601	0.669
A14—Vaginal birth leads to a decrease in sexual satisfaction	0.313			0.758
A16—The baby’s lack of adaptation to life outside the womb is more frequent in cesarean sections			0.584	0.631
A17—Children born by cesarean section are more intelligent		0.708		0.636
A22—Medical interventions (such as induced labor, episiotomy, cesarean section) are excessively used and should be avoided if possible	0.625			0.73

**Table 6:** Consistency analysis of the questionnaire after removing items A7 and A14.

Estimate	McDonald’s ω	Cronbach’s α
Point Estimate	0.714	0.704
Lower Limit of 95% Confidence Interval	0.673	0.660
Upper Limit of 95% Confidence Interval	0.755	0.744

**Table 7:** Item correlations with final score after removing items A7 and A14.

Item	Item-Rest Correlation
A1—Childbirth is a physiological process that should only be intervened in when medically necessary	0.487
A2—Skin-to-skin contact, mother and child, is easier to perform during vaginal birth	0.305
A3—After a vaginal birth, the mother’s recovery is easier and faster	0.405
A4—Vaginal birth increases a woman’s self-confidence	0.535
A5—The emotional bond between mother and child is stronger after a vaginal birth	0.457
A6—Scheduled cesarean sections are associated with the risk of prematurity for the newborn	0.241
A8—Fear of birth pain influences the choice of delivery method	0.533
A9—Elective cesarean sections are more accessible in private healthcare	0.517
A13—The medications used for anesthesia in cesarean sections are harmful to the fetus	0.317
A16—The baby’s lack of adaptation to life outside the womb is more frequent in cesarean sections	0.396
A17—Children born by cesarean section are more intelligent	0.249
A22—Medical interventions (such as induced labor, episiotomy, cesarean section) are excessively used and should be avoided if possible	0.230

**Table 8:** Item loadings on three factors.

Item	Factor			
	1	2	3	Uniqueness
A1—Childbirth is a physiological process that should only be intervened in when medically necessary		0.495		0.649
A2—Skin-to-skin contact, mother and child, is easier to perform during vaginal birth	0.482			0.757
A3—After a vaginal birth, the mother’s recovery is easier and faster	0.501			0.670
A4—Vaginal birth increases a woman’s self-confidence	0.666			0.381
A5—The emotional bond between mother and child is stronger after a vaginal birth	0.701			0.458
A6—Scheduled cesarean sections are associated with the risk of prematurity for the newborn			0.471	0.773
A8—Fear of birth pain influences the choice of delivery method		0.585		0.546
A9—Elective cesarean sections are more accessible in private healthcare		0.461		0.589
A13—The medications used for anesthesia in cesarean sections are harmful to the fetus			0.630	0.656
A16—The baby’s lack of adaptation to life outside the womb is more frequent in cesarean sections			0.574	0.622
A17—Children born by cesarean section are more intelligent		0.753		0.561
A22—Medical interventions (such as induced labor, episiotomy, cesarean section) are excessively used and should be avoided if possible	0.604			0.728

**Table 9:** Factor characteristics.

		Unrotated Solution			Rotated Solution		
	Eigenvalues	Sum of Squared Loadings	Variance Proportion	Cumulative	Sum of Squared Loadings	Variance Proportion	Cumulative
Factor 1	4.789	4.387	0.366	0.366	2.741	0.228	0.228
Factor 2	1.735	1.290	0.107	0.473	2.256	0.188	0.416
Factor 3	1.435	0.926	0.077	0.550	1.605	0.134	0.550

**Table 10:** Indicator estimates for CFA with 95% confidence interval.

						95% Confidence Interval		
Factor	Indicator	Estimate	Std. Error	z-Value	*p*	Lower	Upper	Std. Est. (All)
Factor 1	A2	0.595	0.026	23.204	<0.001	0.544	0.645	0.595
	A3	0.746	0.020	36.862	<0.001	0.706	0.785	0.746
	A4	0.971	0.011	91.416	<0.001	0.951	0.992	0.971
	A5	0.851	0.014	58.684	<0.001	0.822	0.879	0.851
	A22	0.505	0.029	17.527	<0.001	0.449	0.562	0.505
Factor 2	A1	0.706	0.027	26.036	<0.001	0.653	0.759	0.706
	A8	0.688	0.021	32.587	<0.001	0.647	0.730	0.688
	A9	0.790	0.024	32.871	<0.001	0.743	0.838	0.790
	A17	0.444	0.030	14.704	<0.001	0.385	0.503	0.444
Factor 3	A6	0.382	0.042	9.141	<0.001	0.300	0.464	0.382
	A13	0.605	0.026	23.544	<0.001	0.554	0.655	0.605
	A16	0.826	0.029	28.362	<0.001	0.769	0.883	0.826

**Table 11:** Factor estimates with standard errors and 95% confidence interval.

Confidence Interval 95%					
Factor	Estimate	Std. Error	Lower	Upper	Std. Est. (All)
Factor 1	1.000	0.000	1.000	1.000	1.000
Factor 2	1.000	0.000	1.000	1.000	1.000
Factor 3	1.000	0.000	1.000	1.000	1.000

**Table 12:** Factor correlations with 95% confidence interval.

							95% Confidence Interval		
			Estimate	Std. Error	z-Value	*p*	Lower	Upper	Std. Est. (All)
Factor 1	↔	Factor 2	0.763	0.020	38.724	<0.001	0.725	0.802	0.763
Factor 1	↔	Factor 3	0.562	0.031	18.221	<0.001	0.502	0.622	0.562
Factor 2	↔	Factor 3	0.616	0.032	19.175	<0.001	0.553	0.679	0.616

**Table 13:** Estimates of indicators on perceptions of vaginal birth and cesarean section with 95% confidence interval.

95% Confidence Interval					
Indicator	Estimate	Std. Error	Lower	Upper	Std. Est. (All)
A2—Skin-to-skin contact, mother and child, is easier to perform during vaginal birth	0.646	0.000	0.646	0.646	0.646
A3—After a vaginal birth, the mother’s recovery is easier and faster	0.444	0.000	0.444	0.444	0.444
A4—Vaginal birth increases a woman’s self-confidence	0.056	0.000	0.056	0.056	0.056
A5—The emotional bond between mother and child is stronger after a vaginal birth	0.276	0.000	0.276	0.276	0.276
A22—Medical interventions (such as induced labor, episiotomy, cesarean section) are excessively used and should be avoided if possible	0.744	0.000	0.744	0.744	0.744
A1—Childbirth is a physiological process that should only be intervened in when medically necessary	0.501	0.000	0.501	0.501	0.501
A8—Fear of birth pain influences the choice of delivery method	0.526	0.000	0.526	0.526	0.526
A9—Elective cesarean sections are more accessible in private healthcare	0.375	0.000	0.375	0.375	0.375
A17—Children born by cesarean section are more intelligent	0.803	0.000	0.803	0.803	0.803
A6—Scheduled cesarean sections are associated with the risk of prematurity for the newborn	0.854	0.000	0.854	0.854	0.854
A13—The medications used for anesthesia in cesarean sections are harmful to the fetus	0.634	0.000	0.634	0.634	0.634
A16—The baby’s lack of adaptation to life outside the womb is more frequent in cesarean sections	0.317	0.000	0.317	0.317	0.317

**Table 14:** Chi-square test results for baseline and factor models.

Model	Χ^2^	df	*p*
Baseline model	15,070.05	66	
Factor model	230.578	51	<0.001

**Table 15:** Fit indices for model evaluation.

Index	Value
Comparative Fit Index (CFI)	0.988
Tucker–Lewis Index (TLI)	0.985
Bentler–Bonett Non-Normed Fit Index (NNFI)	0.985
Bentler–Bonett Normed Fit Index (NFI)	0.985
Parsimony Normed Fit Index (PNFI)	0.761
Bollen’s Relative Fit Index (RFI)	0.980
Bollen’s Incremental Fit Index (IFI)	0.988
Relative Non-Centrality Index (RNI)	0.988

**Table 16:** Additional fit indices for model evaluation.

Metric	Value
Root mean square error of approximation (RMSEA)	0.052
RMSEA 90% CI lower bound	0.045
RMSEA 90% CI upper bound	0.059
RMSEA *p*-value	0.300
Standardized root mean square residual (SRMR)	0.059
Hoelter’s critical N (α = 0.05)	388.157
Hoelter’s critical N (α = 0.01)	437.302
Goodness of fit index (GFI)	0.989
McDonald fit index (MFI)	0.933

**Table 17:** Factor loadings for the single-factor model.

						95% Confidence Interval	
Factor	Indicator	Estimate	Std. Error	z-Value	*p*	Lower	Upper
Factor 1	A1—Childbirth is a physiological process that should only be intervened in when medically necessary	0.624	0.016	38.655	<0.001	0.592	0.655
	A2—Skin-to-skin contact, mother and child, is easier to perform during vaginal birth	0.566	0.015	37.627	<0.001	0.537	0.596
	A3—After a vaginal birth, the mother’s recovery is easier and faster	0.727	0.014	50.513	<0.001	0.698	0.755
	A4—Vaginal birth increases a woman’s self-confidence	0.943	0.013	72.028	<0.001	0.917	0.969
	A5—The emotional bond between mother and child is stronger after a vaginal birth	0.833	0.012	66.982	<0.001	0.808	0.857
	A6—Scheduled cesarean sections are associated with the risk of prematurity for the newborn	0.290	0.018	16.095	<0.001	0.255	0.326
	A8—Fear of birth pain influences the choice of delivery method	0.619	0.013	47.819	<0.001	0.593	0.644
	A9—Elective cesarean sections are more accessible in private healthcare	0.709	0.014	49.617	<0.001	0.681	0.737
	A13—The medications used for anesthesia in cesarean sections are harmful to the fetus	0.441	0.014	31.199	<0.001	0.414	0.469
	A16—The baby’s lack of adaptation to life outside the womb is more frequent in cesarean sections	0.573	0.015	37.961	<0.001	0.543	0.602
	A17—Children born by cesarean section are more intelligent	0.396	0.016	24.526	<0.001	0.364	0.427
	A22—Medical interventions (such as induced labor, episiotomy, cesarean section) are excessively used and should be avoided if possible	0.475	0.016	30.237	<0.001	0.444	0.505

**Table 18:** Factor variance estimates with 95% confidence interval.

95% Confidence Interval				
Factor	Estimate	Std. Error	Lower	Upper
Factor 1	1.000	0.000	1.000	1.000
**95% Confidence Interval**				
**Indicator**	**Estimate**	**Std. Error**	**Lower**	**Upper**
A1—Childbirth is a physiological process that should only be intervened in when medically necessary	0.611	0.000	0.611	0.611
A2—Skin-to-skin contact, mother and child, is easier to perform during vaginal birth	0.679	0.000	0.679	0.679
A3—After a vaginal birth, the mother’s recovery is easier and faster	0.472	0.000	0.472	0.472
A4—Vaginal birth increases a woman’s self-confidence	0.111	0.000	0.111	0.111
A5—The emotional bond between mother and child is stronger after a vaginal birth	0.307	0.000	0.307	0.307
A6—Scheduled cesarean sections are associated with the risk of prematurity for the newborn	0.916	0.000	0.916	0.916
A8—Fear of birth pain influences the choice of delivery method	0.617	0.000	0.617	0.617
A9—Elective cesarean sections are more accessible in private healthcare	0.497	0.000	0.497	0.497
A13—The medications used for anesthesia in cesarean sections are harmful to the fetus	0.805	0.000	0.805	0.805
A16—The baby’s lack of adaptation to life outside the womb is more frequent in cesarean sections	0.672	0.000	0.672	0.672
A17—Children born by cesarean section are more intelligent	0.843	0.000	0.843	0.843
A22—Medical interventions (such as induced labor, episiotomy, cesarean section) are excessively used and should be avoided if possible	0.775	0.000	0.775	0.775

**Table 19:** Chi-square test results for baseline and factor models.

Model	Χ^2^	df	*p*
Baseline model	15,070.054	66	
Factor model	586.490	54	<0.001

**Table 20:** Model fit indices.

Index	Value
Comparative Fit Index (CFI)	0.965
Tucker–Lewis Index (TLI)	0.957
Bentler–Bonett Non-Normed Fit Index (NNFI)	0.957
Bentler–Bonett Normed Fit Index (NFI)	0.961
Parsimony Normed Fit Index (PNFI)	0.786
Bollen’s Relative Fit Index (RFI)	0.952
Bollen’s Incremental Fit Index (IFI)	0.965
Relative Non-Centrality Index (RNI)	0.965

**Table 21:** Other model fit indices.

Metric	Value
Root mean square error of approximation (RMSEA)	0.087
RMSEA 90% CI lower bound	0.081
RMSEA 90% CI upper bound	0.094
RMSEA *p*-value	0.000
Standardized root mean square residual (SRMR)	0.081
Hoelter’s critical N (α = 0.05)	160.933
Hoelter’s critical N (α = 0.01)	180.695
Goodness of fit index (GFI)	0.972
McDonald fit index (MFI)	0.815

## Data Availability

The raw data supporting the conclusions of this article will be made available by the authors on request.

## Public Involvement Statement

No public involvement in any aspect of this research.

## Guidelines and Standards Statement

A complete list of reporting guidelines can be accessed via the equator network: https://www.equator-network.org/ (accessed on 1 June 2024).
